# Mortality-Culling Rates of Dairy Calves and Replacement Heifers and Its Risk Factors in Holstein Cattle

**DOI:** 10.3390/ani9100730

**Published:** 2019-09-26

**Authors:** Hailiang Zhang, Yachun Wang, Yao Chang, Hanpeng Luo, Luiz F. Brito, Yixin Dong, Rui Shi, Yajing Wang, Ganghui Dong, Lin Liu

**Affiliations:** 1 Key Laboratory of Animal Genetics, Breeding and Reproduction, MARA, National Engineering Laboratory of Animal Breeding, College of Animal Science and Technology, China Agricultural University, Beijing 100193, China; zhl108@cau.edu.cn (H.Z.); changyao@cau.edu.cn (Y.C.); luohanpeng@cau.edu.cn (H.L.); dyx18634593316@163.com (Y.D.); srandeffy@163.com (R.S.); 2 Department of Animal Sciences, College of Agriculture, Purdue University, West Lafayette, IN 47907, USA; britol@purdue.edu; 3 State Key Laboratory of Animal Nutrition, College of Animal Science and Technology, China Agricultural University, Beijing 100193, China; yajingwang@cau.edu.cn; 4 Beijing Sunlon Livestock Development Co. Ltd, Beijing 100176, China; ganghui89@126.com; 5 Beijing Dairy Cattle Center, Beijing 100192, China; liulin@bdcc.com.cn

**Keywords:** dairy calf, involuntary culling, mortality, replacement heifer, survival rate

## Abstract

**Simple Summary:**

High mortality and involuntary culling rates cause great economic losses to the dairy industry around the world, and the survival of dairy calves and replacement heifers is paramount in modern dairy breeding. However, little has been done to genetically improve mortality rates of dairy calves and replacement heifers in Chinese Holstein cattle. In this study, we investigated population parameters (descriptive statistics) of mortality rates of dairy calves and replacement heifers and risk factors affecting mortality and involuntary culling rates in Chinese Holstein cattle. The mortality rate of dairy calves and replacement heifers from day 3 to 60, 61 to 365, and 366 to first calving was 5.5%, 7.4%, and 8.7%, and an unfavorable increasing trend has been observed in the Chinese Holstein population. Health events associated with digestive and respiratory or circulatory systems were the main reasons for deaths. Herd-birth year, birth season, and dam parity had significant effects on survival. Our findings will help farmers to better manage dairy calves and replacement heifers and highlight the need to include these survival traits as part of the national genetic evaluation schemes.

**Abstract:**

The rates of mortality and involuntary culling of dairy calves and replacement heifers have great economic implications on the dairy cattle industry around the world. The main objectives of this study were: (1) to obtain population parameters of mortality and involuntary culling rates of dairy calves and replacement heifers; and, (2) to investigate the factors affecting mortality and involuntary culling rates in Chinese Holstein cattle. Two datasets containing records of birth, calving, and culling events from 142,833 Holstein cattle born between 1991 and 2018 were used in this study. The population parameters were obtained using dataset 1, which consisted of dairy calves and replacement heifers that died or were involuntarily culled. Three survival traits were defined in dataset 2, which consisted of females born from 1999 to 2018. A binomial logistic regression was used to analyze the risk factors on the survival traits. The mortality rate of dairy calves and replacement heifers from day 3 to 60, 61 to 365, and 366 to first calving was 5.5%, 7.4%, and 8.7%, and an unfavorable increasing trend was observed. Health events associated with digestive and respiratory or circulatory systems were the main death reasons. Herd-birth year, birth season, and dam parity had significant effects on survival traits. The results from this study will help farmers to better manage calves and replacement heifers and highlight the need to include survival traits in dairy calves and replacement heifers as part of national genetic evaluation schemes.

## 1. Introduction

The large majority of dairy cattle herds are divided into two groups: milking cows and replacement heifers, in which the latter does not generate any direct income to the producers until the first calving [1,2]. Estimates of expenses associated with rearing replacement heifers range from 15% to 20% of the total milk production costs [3]. However, many potential replacement heifers do not reach their first lactation due to premature death or involuntary culling [2]. Therefore, in addition to welfare issues, high mortality and culling rates cause great economic losses to the dairy industry around the world [4,5]. Various breeding programs include indicator traits of health and longevity measured in dairy cows [6]. However, less importance has been given to survivability of dairy calves and replacement heifers. 

The mortality rates of calves and replacement heifers vary across countries, production systems, and populations. For instance, in the United States, annual calf and heifer mortality rates were estimated to be around 9.6%, with pre-weaning calves accounting for 7.8% [7]. In Danish Holstein, the frequency of pre-pubertal mortality was estimated to be 5% to 6% [8], and the most frequent diseases affecting calves were diarrhea and respiratory diseases. Other studies showed that the main causes of replacement heifer mortality or involuntary culling were different depending on the life stage of the animal. Scours, diarrhea, and other digestive problems were the most important death causes of pre-weaning calves, while respiratory diseases were the largest death cause of weaned calves [9]. Furthermore, many herd- or animal-level risk factors affecting dairy calf and heifer mortality have been identified in various populations. These factors include dystocia, sex, twinning rate, dam parity, herd size, and birth season [10,11,12,13]. 

The impacts of mortality rates of calf and replacement heifer on dairy cattle herds should not be neglected. However, there is a lack of literature reporting on this issue in calves and replacement heifers in Chinese Holstein population, especially studies based on individual records. In this context, the main objectives of this study were: (1) to estimate population parameters (descriptive statistics) of mortality and involuntary culling rates of dairy calves and replacement heifers; and, (2) to investigate the risk factors affecting mortality and involuntary culling of dairy calves and heifers in Chinese Holstein cattle, using individual records. The findings of this study will help farmers to design better management strategies for the dairy calves and replacement heifers, and provide a reference for further investigation on the genetic background of survival traits in dairy calves and replacement heifers.

## 2. Materials and Methods 

The records of birth, calving and culling/death events from 1999 to 2018 in female Holstein from 31 herds located in Beijing, Tianjin, Yunnan, Hebei, Henan, Heilongjiang, Jilin, and Inner Mongolia were extracted from the farm management software (AfiFarm, http://www.afimilk.com.cn). The free-stall barn system was used in all herds, and the herds’ sizes ranged from 1000 to 10,000 animals. The test-day milk yield in these herds ranged from 30 kg to 40 kg. The herd records before using management software (before 2005) were added into software from herdbook records, and thus, early records might be incomplete or less accurate. Two datasets were defined using event records: Dataset 1 and dataset 2. Dataset 1 included records of dairy calves and replacement heifers that left herds (before the first calving) between 2006 and 2018, which was used to obtain population parameters of involuntary culling/death age on dairy calves and replacement heifers in Chinese Holstein cattle, including average involuntary culling/death age and culling/death reason. The dataset 2 included records from all animals (that left herds either before or after first calving) born from 1999 to 2018, which was only used to investigate risk factors affecting survivability of dairy calves and replacement heifers using logistic regression. In Chinese Holstein herds, most calves left the herd due to premature death, while heifers can also be culled for reproduction disorders, severe disease and other reasons. In this study, we are interested in both mortality and involuntary culling. The records of calves that died within 2 days after birth, replacement heifers that died after 1800 days of age (60 months) and censored records (alive dairy calves and replacement heifers, and sold and transferred individuals) were removed from both dataset 1 and dataset 2. The death records before first 48 h were considered as stillbirth, which is usually a separate dam trait and thus is not part of the current study. In dataset 1, the involuntary culling/death reasons were grouped in a total of 10 categories: digestive system diseases, diseases of respiratory or circulatory systems, reproduction disorders, death without clear reasons, infectious diseases, developmental disorders (e.g., abnormalities and dysplasia), feet and leg diseases, accidental injury, other diseases (e.g., septicemia and meningitis) and unknown reason. Only females were kept in the datasets. After editing, records for 18,077 culled dairy calves and replacement heifers remained in dataset 1 and 113,218 records of all animals in dataset 2. Death/culling age (days) was calculated for each animal in dataset 1 and referred to the interval from birth to death/culling on both dairy calves and replacement heifers. A total of 3 survival traits were defined for females in dataset 2, including survival from 3 to 60 days (Sur1), 61 to 365 days (Sur2), and from day 366 to first calving (Sur3). Survival traits were analyzed as binary traits, in which a value of “0” was assigned to animals that left the herds and “1” to those that survived up to next life stage.

A binomial logistic regression was used to evaluate the risk factors affecting survivability of dairy calves and replacement heifers using the LOGISTIC procedure of SAS software (version 9.1; SAS Institute, 2004 [14]). A total of 4 risk factors associated with survival traits were analyzed using the dataset 2. These factors were herd-birth year (402 levels), birth season (divided into Spring: March to May, Summer: June to August, Fall: September to November, and Winter: December to February), dam parity (defined as 0 = unknown, 1 = first parity, 2 = second parity, 3 = third and greater parities), calving ease score (defined as 0 = unknown, 1 = unassisted, 2 = easy pull, and 3 = hard pull or surgery). The factor of herd-birth year represented the combined effect of herd and birth year of calf, and 402 levels were combined into 5 levels using logit (*p*) of each level. The statistical model for Sur1, Sur2, and Sur3 can be described as follow:

$$\mathrm{logit}\left(p\right)=\ln\left(\frac{p}{1-p}\right)=\;\beta_{0}+\;\beta_{1}x_{1}+\;\beta_{2}x_{2}\;+\;\beta_{3}x_{3}\;+\;\beta_{4}x_{4},$$

where *p* is the culling probability of dairy calves and replacement heifers in each life stage; $\beta_{0}$ is the overall mean (intercept); $\beta_{1}$ to $\beta_{4}$ are the regression coefficients of ranked factors; $x_{1}$, $x_{2}$, $x_{3}$, and $x_{4}$ correspond to the herd-birth year, birth season, dam parity and calving ease score, respectively, associated with each observation.

## 3. Results

### 3.1. Descriptive Statistics

#### 3.1.1. Mortality-Culling Frequency of Dairy Calves and Replacement Heifers

**Figure 1:**
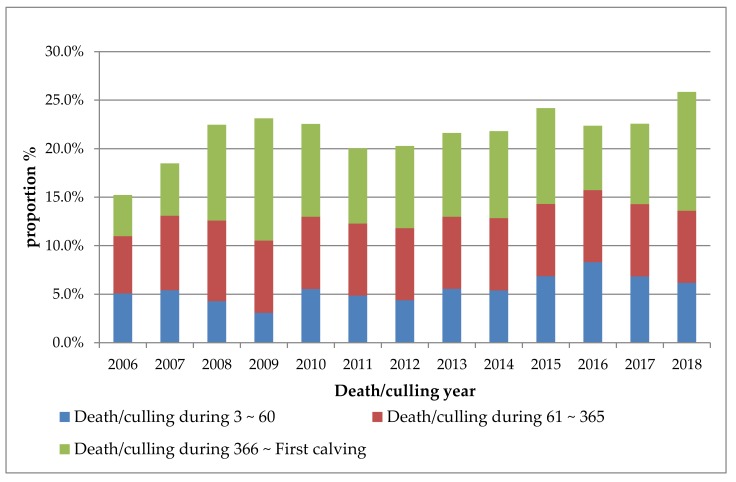
Mortality-culling rates of dairy calves and replacement heifers in different life stages over time.

The combined mortality-culling rate of female dairy calves and replacement heifers was 21.2%, in which the mortality-culling rate from day 3 to 60, 61 to 365, and 366 to first calving was 5.5%, 7.4%, and 8.7%, respectively. The variability in mortality-culling within each life stage over time is shown in Figure 1. From 2006 to 2008, the mortality-culling rate of dairy calves and replacement heifers increased from 15.2% to 25.9% (an increase rate of 70.4%). 

#### 3.1.2. Death/Culling Age of Dairy Calves and Replacement Heifers

**Figure 2:**
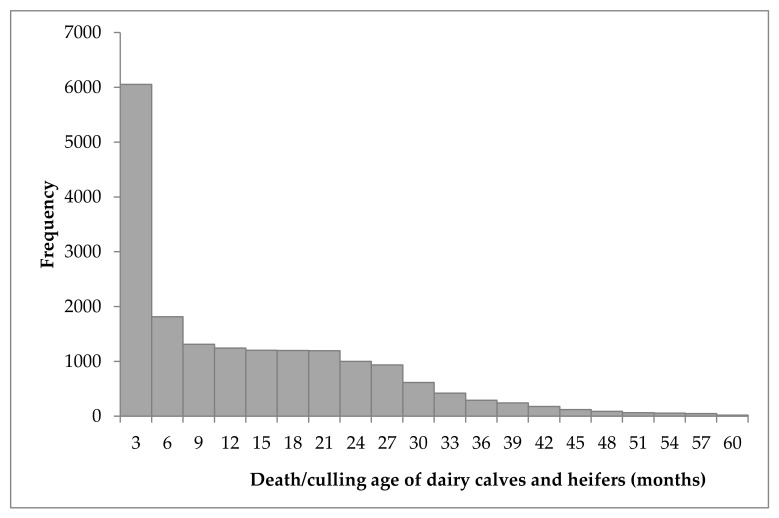
Histogram of death/culling age (months) on dairy calves and replacement heifers.

**Figure 3:**
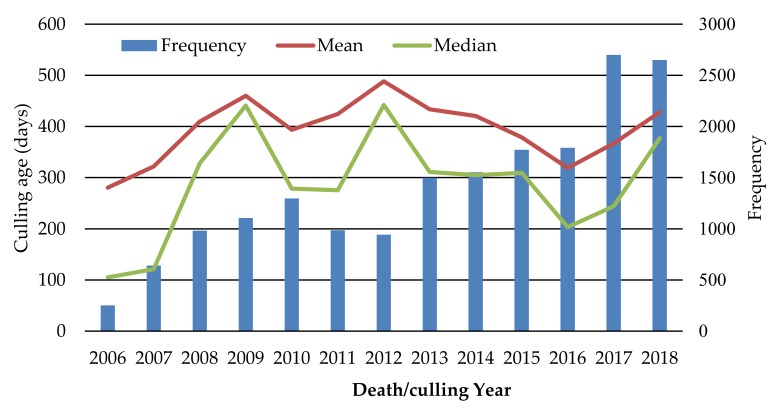
Average death/culling age (days) and number of dairy calves and replacement heifers in different death years.

Descriptive statistics of death/culling age for 18,077 dairy calves and replacement heifers born from 2006 to 2018 were calculated. The average death/culling age was 399 days; the median 296 days and the lower and upper quartile were equal to 84 and 658 days, respectively. The death/culling age did not follow a normal distribution as the large majority of calves died early in life (Figure 2). The highest mortality-culling risk on dairy calves was within the first 100 days after birth. Mortality-culling tended to decrease with the increase of animal age. The mean and median of death/culling age over time are presented in Figure 3. Over the past 13 years, there was a large difference on death/culling age of dairy calves and replacement heifers and the average death/culling age fluctuated around 400 days. Furthermore, the variation range of median death/culling age was larger than the means among different years, and the maximum difference of median death/culling age was 337 days (between 2006 and 2012).

### 3.2. Death/Culling Reasons of Dairy Calves and Replacement Heifers

**Figure 4:**
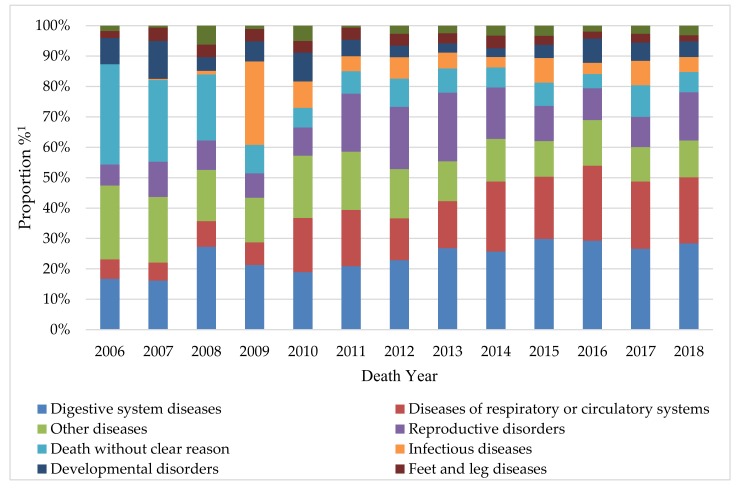
The death proportions of dairy calves and replacement heifers caused by different reason categories over time.^1^ The category of “unknown reason” was not included in Figure 4.

The overall death proportion of each reason category in period from day 3 to first calving in different years is presented in Figure 4. “Unknown reason” was not included in Figure 4, which was reached 8.99%–43.78% over these years. Diseases related to the digestive, respiratory and circulatory systems, and reproductive disorders were the main causes of death. These categories accounted for 58.6% of the known causes of death. Diarrhea, pneumonia, and infertility (based on non-return rate) were the main specific reasons of mortality. Over the past 13 years, the death proportion due to diseases related to the respiratory and circulatory systems and reproductive disorders gradually increased, in contrast with both infectious diseases and other diseases that showed a decrease trend. The average involuntary culling or death ages of dairy calves and replacement heifers based on different death reason categories are presented in Table 1. The individuals with digestive system diseases, diseases of respiratory or circulatory systems, and death without clear reason were culled in early life (mean: up to 226.6 days; median: up to 101.0 days). As expected, the average death age of individuals with reproductive disorders (mean: 937.4; median: 891.0) was greater compared to the other categories (mean range: 165.1–476.7 days; median range: 84.0–450.5 days).

### 3.3. Analyses of the Factors Influencing Survivability of Dairy Calves and Replacement Heifers

According to the Wald test (Chi-square), both herd-birth year and birth season significantly (*p* < 0.01) influenced the mortality of dairy calves and replacement heifers during the stages of 3–60 days (Sur1), 61–365 days (Sur2), and from 366 days to first calving (Sur3). The dam parity significantly influenced Sur1 and Sur2 (*p* < 0.01), and did not significantly influence Sur3 (*p* = 0.19). However, dam calving ease score did not significantly impact any of the 3 survival traits. The results of the binomial logistic regression on Sur1, Sur2, and Sur3 in dairy calves and replacement heifers are presented in Table 2.

The dairy calves and replacement heifers born in Spring had the lowest mortality risk in any of the 3 life stages. Across the 3 life stages (Sur1, Sur2, and Sur3), the mortality risk of dairy calves and replacement heifers born in Fall was between 1.13 and 1.53 times greater than those animals born in the Spring season. The calf birth season had larger impact on survivability of animals during 3–365 days (Sur1 and Sur2) compared to 366 days to first calving. In terms of dam parity, calves born from second parity cows had the lowest culling risk in any of the 3 life stages. From 3 to 60 days, calves born from first parity cows had the highest culling risk, i.e., 1.16 times greater than those animals born from second parity cows. However, the dairy calves and replacement heifers born from cows with 3 or more parities had the highest mortality risk during 61–365 and 366–first calving. Animals born by hard pull or surgery had the highest mortality risk, which was not significant compared with unassisted calves.

## 4. Discussion

The involuntary culling and mortality rates of dairy calves and replacement heifers have been reported to vary across countries and dairy cattle populations. In the population used for the current study, dairy calves were usually weaned at 2 months of age, and the pre-weaning mortality rate was 5.5%, which is within the range reported in the literature. For instance, the calf mortality within the first month of life were 3.1%–3.4% in Danish [15] and UK [2] Holstein populations, while in the US, the mortality of pre-weaning Holstein calves was 7.8% [7]. The mortality rate of 12.9% for dairy calves up to yearling age is also within the ranges reported in the literature for worldwide dairy populations (3.7%–22.5%) [16,17]. Approximately 21.2% of dairy calves and heifers failed to reach first calving, which is substantially higher compared to other reports (e.g., 14.5%) [18]. Furthermore, there was an unfavorable increase trend on mortality of dairy calves and replacement heifers over time in Chinese Holstein population. The calf and replacement heifer survival between day 3 and the start of productive life should be given more attention, especially for genetically select animals with better genetic merit for survival traits.

Many factors have caused mortality of dairy calves and replacement heifers, including calf-related diseases, heifer fertility disorders and farm management factors. In this study, censored and voluntary culling records were removed from the datasets. Therefore, the mortality rates reported here represent involuntary culling in Chinese Holstein calves and replacement heifers. Due to poor data management and insufficient attention paid on data recording, culling/death reason were not always available for each animal, especially in early records. In general, the 2 most frequent causes of mortality are digestive [19,20] and respiratory diseases [21], in which diarrhea and pneumonia accounts for the majority of death cases [9,20,22]. In this study, diseases associated with the digestive, respiratory and circulatory systems were the main culling reasons, which is consistent with the findings reported in other studies. In the US Holstein cattle population, scours, diarrhea, and other digestive problems were the key causes of pre-weaning calf mortality, followed by respiratory diseases. For weaned calves, respiratory disease was the largest mortality reason in the US population [9]. Pritchard et al. [1] reported that a large number of heifers were culled due to been considered unsuitable as breeding replacement, failure to conceive and other reproduction disorders, which is in agreement with our findings. The median death age of calves or replacement heifers due to digestive system diseases, diseases of respiratory or circulatory systems, and reproductive disorders were 84, 101, and 891 days, respectively. Furthermore, the main causes of mortality were different over these years in calves and replacement heifers. The fertility recession and more attention on epidemic prevention may respectively result in increase/decrease trends of culling/death proportion of reproductive disorders/infectious diseases over these years. 

Considering the impacts of management differences across herds and a likely interaction with birth year, the effect of herd was included in the statistical model as a combined effect (herd-birth year) with birth year of the calf in current study. Herd-birth year significantly impacted all survival traits consistent with Norberg et al. [8]. Birth season and dam parity significantly impacted all survival traits, which is in agreement with results reported by Norberg et al. [8] and Gulliksen et al. [16]. During Summer and Fall, animals can be under heat stress in the main dairy farming areas in China (including the herds in the current study). The calves that experience maternal heat stress during late gestation have been reported to have reduced survival rate before puberty [23]. Dairy producers can plan the calving accordingly in order to reduce calving mortality rates and/or implement other mitigation approaches. Furthermore, Henderson et al. [24] and Ring et al. [25] reported that dam calving ease score was an important risk factor of mortality, especially within the first 182 days of life. According to them, the calves and heifers born from increased calving ease score were more likely to die in early life stages. This is likely due to the stress suffered by calves during birth. The dam calving ease score had no statistically significant impact on the survival traits analyzed here, which may be related a small data size in current study. These information from risk factors will help farmers to reduce mortality rate of calves by implementing better management practices in their herds. In addition, the influence of the risk factors identified here will be important effects to be included in the statistical models for genetic evaluation for survival traits in dairy calves and replacement heifers.

Survival traits defined at different life periods during replacement heifer development may enable selection against certain diseases commonly prevalent during those life stages [1]. Three survival traits were defined in this study aiming to describe the likelihood of death (or survival) during pre-weaning period, day 61 to yearling and yearling to the first calving. Survival trait at early life of the calf (Sur1) may enable indirect selection against diseases related to the digestive, respiratory, and circulatory systems, which were the two most frequent mortality reason categories. The survival traits at different life stages, defined in this study, can be used to genetically improve the mortality rates of dairy calf and replacement heifers, and the results from the current study laid the foundation for establishing the statistical models for genetic evaluations. The next step will be the estimation of genetic parameters (heritability and genetic correlations) for the survival traits defined in this study.

## 5. Conclusions

The combined mortality rate of dairy calves and replacement heifers in Chinese Holstein cattle was 21.2% and an unfavorable trend on dairy calves and replacement heifer’ mortality was observed. Diseases related with digestive (e.g., diarrhea), respiratory (e.g., pneumonia) and circulatory systems, and reproductive disorders (infertility based on non-return rate) were the main death reason categories. Herd-birth year, birth season, and parity of dam had significant effects on the survival traits of dairy calves and replacement heifers. Survival traits in dairy cattle from birth to first calving are important breeding goals to be incorporated into dairy genetic selection schemes.

## Figures and Tables

**Table 1:** The death age (days) of dairy calves and replacement heifers caused by different reason categories.

Death Reasons	Proportion (%)	Death Age (Days)				
		Mean	Median	Lower Quartile	Upper Quartile	SD
Unknown reason	26.0	449.8	430.0	259.0	638.0	262.3
Digestive system diseases	18.9	226.6	84.0	20.0	227.0	350.3
Diseases of respiratory or circulatory systems	13.6	165.1	101.0	51.3	173.0	200.9
Reproductive disorders	10.3	937.4	891.0	776.0	1085.0	259.1
Death without clear reason	6.9	200.6	90.0	3.0	234.0	264.4
Infectious diseases	4.8	444.1	437.0	237.0	635.0	250.4
Developmental disorders	4.2	396.9	413.0	67.3	601.5	334.3
Feet and leg diseases	2.4	470.8	413.5	98.0	743.5	400.7
Accidental injury	2.1	476.7	450.5	266.0	668.5	289.9
Other diseases	10.8	417.1	310.0	82.0	678.0	390.7

**Table 2:** Associations between different levels of birth season, dam parity and calving ease score on the odds ratio of mortality ^1^.

Variable	Level	Odds Ratio	95% Confidence Interval		*p*-Value
Survival from 3 to 60 days					
Birth season	Summer vs Spring	1.48	1.34	1.64	<0.01
	Autumn vs Spring	1.56	1.41	1.73	<0.01
	Winter vs Spring	1.60	1.44	1.77	<0.01
Dam parity	Unknown vs 2	0.45	0.28	0.71	<0.01
	1 vs 2	1.16	1.02	1.33	0.03
	“3 and above” vs 2	1.14	0.98	1.32	0.09
Calving ease score	Unknown vs 1	1.46	0.93	2.30	0.10
	2 vs 1	1.39	0.97	1.99	0.07
	3 vs 1	1.83	0.77	4.34	0.17
Survival from 61 to 365 days					
Birth season	Summer vs Spring	1.53	1.41	1.65	<0.01
	Autumn vs Spring	1.64	1.52	1.77	<0.01
	Winter vs Spring	1.18	1.08	1.28	<0.01
Dam parity	Unknown vs 2	0.54	0.33	0.87	0.01
	1 vs 2	1.02	0.93	1.12	0.68
	“3 and above” vs 2	1.13	1.01	1.25	0.03
Calving ease score	Unknown vs 1	1.00	0.62	1.60	0.98
	2 vs 1	0.87	0.61	1.23	0.42
	3 vs 1	1.36	0.67	2.76	0.39
Survival from 366 to first calving					
Birth season	Summer vs Spring	1.13	1.06	1.21	<0.01
	Autumn vs Spring	1.08	1.01	1.16	0.02
	Winter vs Spring	1.08	1.01	1.15	0.03
Dam parity	Unknown vs 2	0.90	0.52	1.57	0.72
	1 vs 2	1.04	0.95	1.14	0.39
	“3 and above” vs 2	1.11	1.01	1.23	0.04
Calving ease score	Unknown vs 1	0.75	0.43	1.30	0.31
	2 vs 1	0.99	0.72	1.36	0.95
	3 vs 1	1.20	0.60	2.40	0.61

^1^ The Spring season, second parity and calving ease score 1 were the base classes of birth season, dam parity and dam calving ease score, respectively. The results of herd-birth year are not shown.
